# Bimodal distribution of intestinal *Candida* in children with autism and its potential link with worse ASD symptoms

**DOI:** 10.1080/29933935.2024.2358324

**Published:** 2024-06-27

**Authors:** Khemlal Nirmalkar, Jigar Patel, Dae-Wook Kang, Andrew Bellinghiere, Devin A. Bowes, Fatir Qureshi, James B. Adams, Rosa Krajmalnik-Brown

**Affiliations:** a Biodesign Center for Health Through Microbiomes, Arizona State University, Tempe, AZ, USA; b Whitehead Institute for Biomedical Research, Cambridge, MA, USA; c School for Engineering of Matter, Transport, and Energy, Arizona State University, Tempe, AZ, USA; d School of Sustainable Engineering and the Built Environment, Arizona State University, Tempe, AZ, USA

**Keywords:** Autism spectrum disorder (ASD), internal transcribed spacer (ITS) sequencing, mycobiota, fungal microbiota, candida albicans

## Abstract

The gastrointestinal (GI) tract harbors a complex and remarkably diverse microbial ecosystem that profoundly impacts various aspects of health and pathophysiology. While bacteria overwhelmingly represent most of the GI microbiota, it is imperative to consider the presence and function of fungal constituents (i.e. mycobiota) within the GI ecosystem. The substantial incidence of GI disorders and associated manifestations in children diagnosed with autism spectrum disorder (ASD) suggests a plausible contributory role of the gut mycobiota. This work aimed to elucidate the gut mycobiota in a cohort of 38 typically developing children (TD) and 40 children with ASD. Fecal samples were collected from all participants, autism severity and GI symptoms were assessed to unravel the potential implications of mycobiota alterations in the gut. We performed fungal internal transcribed spacer (ITS) gene amplicon sequencing to analyze the fungal composition and investigate their relationship with GI and autism symptoms. Among gut mycobiota, *Saccharomyces cerevisiae* was significantly lower (relative abundance) in the ASD fecal samples compared to TD children. *Candida* and *C. albicans* demonstrated a bimodal distribution among children with ASD. The small subset of children with elevated *C. albicans* or decreased *S. cerevisiae* had increased Autism Treatment Evaluation Checklist (ATEC) scores. Our findings suggest that a deficit of *S. cerevisiae*, and an overgrowth of *C. albicans* in a subset of children is associated with worse autism severity. Future work employing shotgun metagenomics with a larger cohort is encouraged to advance understanding of the functional role of fungi, and their possible interplay with GI symptoms and autism severity in children with ASD.

## Introduction

The incidence of autism spectrum disorder (ASD) in the United States has been steadily increasing, and recent reports by the Centers for Disease Control and Prevention (CDC) report 1 in 36 children are diagnosed with ASD.^1^ ASD is a major neurodevelopmental disorder with impairment in social communication, repetitive and restricted behavior.^2,3^ The etiology of ASD is not well understood but it is a multifactorial neurodevelopmental condition influenced by genetic and environmental factors.^2,3^ Moreover, ASD is frequently associated with gastrointestinal (GI) disorders^3–7^ and the prevalence of GI issues in ASD varies, ranging from 4.2%-96.8% but the general estimate is approximately 33–46%.^8,9^ GI issues in ASD individuals typically emerge in early infancy.^4,10^ Notably, the severity of ASD correlates with the severity of GI issues, and it’s associated with gut microbiota.^4,11^ It has been reported that dysbiosis in the gut microbiota composition,^10–12^ alteration in functional genes abundance or expression^12^ such as those that encode neurotransmitters,^13^ microbial detoxification,^14^ amino acid, and xenobiotic metabolism^3^ can disrupt the gut-brain axis, and may contribute to the etiology of ASD.^15–17^

An imbalance in the gut bacterial composition is present in children with ASD and is linked with ASD and gastrointestinal (GI) symptoms.^11^ Common gut bacteria, such as *Bifidobacterium*, and *Prevotella*, have been found in lower abundance,^10,15,18,19^ however in some studies, increased abundance of *Prevotella*^20,21^ and potentially pathogenic bacteria such as *Clostridia, Sutterella*, and *Bacteroides* have been observed in children with ASD compared to typically developing (TD) controls.^22–27^

Abnormal gut bacteria can result from increased oral antibiotic usage, a common occurrence in children with ASD.^10,11,28^ This combined scenario of excessive antibiotic usage and abnormal gut bacteria can lead to the overgrowth of opportunistic intestinal fungi in ASD.^29^ An increased abundance of fungi (primarily *Candida*) has been reported in 25–58% of children with ASD, at rates substantially higher than in TD individuals. ^4,30–34^ Among fungi, a higher relative abundance of *Aspergillus* was reported in one ASD child (a case report),^35^ but in contrast, a lower relative abundance of *Aspergillus versicolor* and a higher relative abundance of *Saccharomyces* was found in 29 ASD children compared to 31 TD.^32^

*Candida* has been associated with ASD, presumably due to the production of mycotoxins, such as alcohols and aldehyde,^36–40^ known to adversely impact mental function^40^ potentially exacerbating existing ASD-related symptoms.^41^ It was hypothesized that the increase in yeast in ASD children is associated with lower secretory Immunoglobulin A (sIgA),^4^ which is the primary antibody combating bacteria and fungi in the mucous membranes.^42,43^ A small open-label clinical trial revealed that treating yeast infections with antifungals led to some clinical improvements in symptoms,^41^ and a national survey of over 27,000 ASD families found that anti-fungal treatments (Nystatin and Diflucan) were rated among the most effective treatments for ASD symptoms.^44^ Only a limited number of studies have reported fungi in ASD via culturing^4,30,31^ or sequencing techniques^32–34^ but most did not investigate the possible correlation of fungi with GI or ASD symptoms, except Alookaran *et al*., who investigated the association between fungi and GI and found no link between them.^33^ It is important to explore the potential link between fungal microbiota and ASD in children, whether all ASD children exhibit a higher prevalence or abundance of fungi, and whether the severity of GI or autism symptoms is linked with fungi abundance.

To address this critical knowledge gap, the current pilot study investigates the gut mycobiota in children with ASD and TD, using fungal ITS gene amplicon sequencing and qPCR targeting the fungal 18S rRNA gene. We analyzed differences in fungal composition and their relationship with GI and ASD symptoms.

## Results

### Positive correlation was observed between worse GI issues and autism symptoms

**Figure 1.**
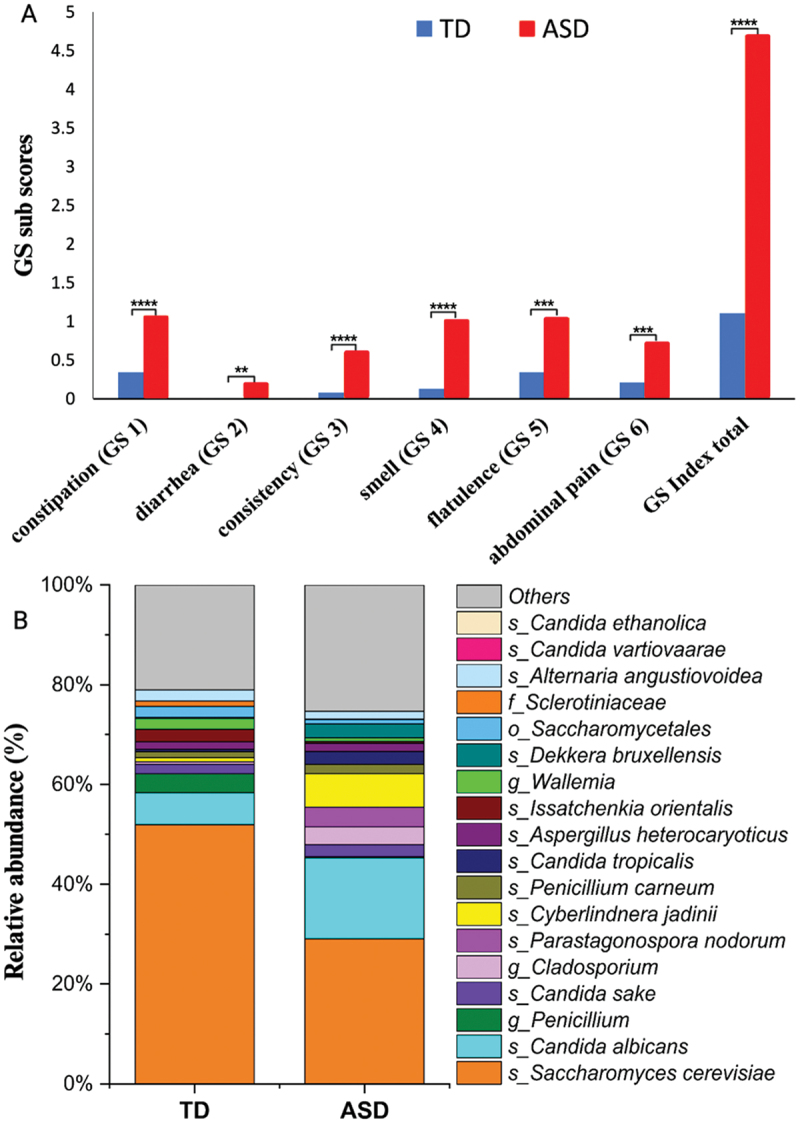
Univariate comparison of gastrointestinal (GI) sub-scores between TD and ASD children (A), and an average relative abundance of most dominant fungal microbiota in TD and ASD children (B). For the relative abundance of fungi in each participant, refer to supplementary figure S4. Blue color represents TD and red color for ASD participants. TD- typically developing; ASD- autism spectrum disorder; GS- Gastrointestinal severity scores; o-order; f-family; g-genera; s-species. *Single asterisk indicates *p* < 0.05, **double asterisks indicate *p* < 0.01, triple ***asterisks indicate *p* < 0.001 and four ****indicate *p* < 0.0001.

We evaluated GI symptoms with six sub-items of the 6-GSI (6-Item Gastro-Intestinal Severity Index): constipation (GS1), diarrhea (GS1), consistency (GS1), smell (GS1), flatulence (GS1), and abdominal pain (GS1) and total GSI in TD and ASD children. As seen in Figures 1a, children in the ASD cohort exhibited significantly higher (*p* < 0.05) GSI symptoms in comparison to their TD counterparts. The relationship between total 6-GSI and Autism Treatment Evaluation Checklist (ATEC) score in children with ASD, revealed a significantly moderate-positive correlation (Spearman *R* = 0.37, *p* = 0.02; Pearson *R* = 0.36, *p* = 0.02) (Figure S1) indicating that in this cohort autism symptoms were linked with GI symptoms.

### *S.* *cerevisiae* and *C.* *albicans* dominated the cohort

**Figure 2.**
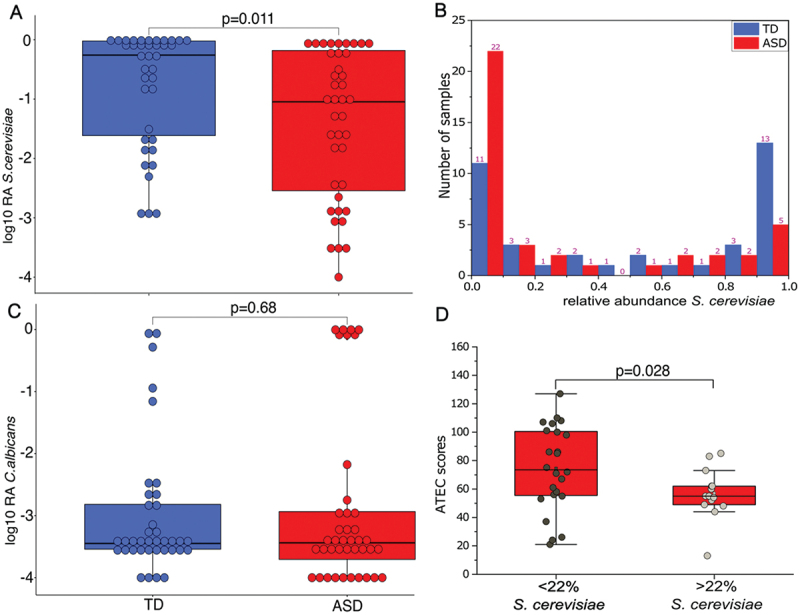
Univariate comparison of the relative abundance (after log10 transformation) of *S. cerevisiae* (A), and distribution (B), univariate comparison of the relative abundance (after log10 transformation) of *C. albicans* (C) in TD and ASD participants. ATEC scores comparison between < 22% and > 22% relative abundance of *S. cerevisiae* in children with ASD (D). TD- typically developing; ASD- autism spectrum disorder. Blue color represents TD and red color for ASD participants. *p*-values <0.05 are considered statistically significant and taxa *p*-values are adjusted with the leave-one-out correction method. Relative abundance scale 0–1 = 0–100%; ATEC scale 0–140; horizontal line inside the box represents the median.

After finding the differences in GI symptoms, we evaluated the fungal microbiota composition using ITS fungal gene amplicon sequencing of fecal DNA from 38 TD and 40 ASD children (Table S1), where we identified 5,089,812 reads, averaging 65,254 reads per sample. Qiime2^45^ and downstream analyses (refer to methods) did not exhibit any significant difference in alpha (Figure S2) and beta diversity (Figure S3) between TD and ASD children. With the UNITE database,^46^ we identified 146 fungal taxa, in which *S.* *cerevisiae*, *C.* *albicans, Penicillium, Candida sake, Candida tropicalis, Aspergillus heterocaryoticus, Cladosporium* were the most abundant fungi (Table S2). Figure 1b illustrates fungi taxonomic relative abundance per cohort and Figure S4 for each child. S. *cerevisiae* was more dominant in the gut mycobiota of TD, whereas *C.* *albicans* was dominant in the ASD group (Figure 1b). Notably, *S.* *cerevisiae* was significantly lower (adjusted *p* < 0.05) in ASD participants compared to TD group (Figure 2a). In contrast, *C.* *albicans* did not exhibit a statistically significant difference (raw *p* > 0.05) between ASD and TD (Figure 2c).

### *S.* *cerevisiae* and *C.* *albican*s exhibited a bimodal distribution

**Figure 3.**
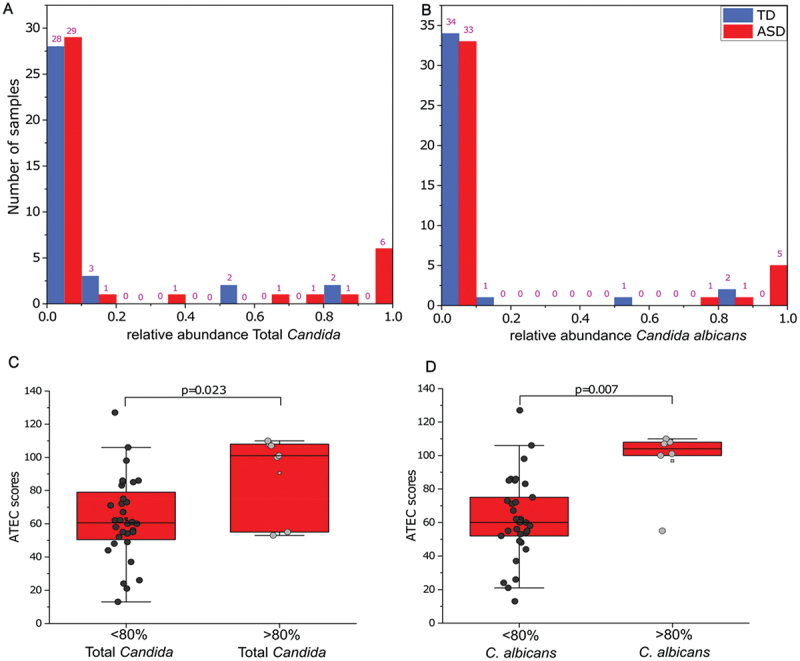
Distribution of total *Candida* (A) and *C. albicans* (B) relative abundance in TD and ASD participants. ATEC scores comparison between < 80% and > 80% relative abundance of *Candida* (C) and *C. albicans* (D) in children with ASD. Blue color represents TD and red color for ASD participants. TD- typically developing; ASD- autism spectrum disorder. *p* values < 0.05 are considered statistically significant. Relative abundance scale 0–1 = 0–100%; ATEC scale 0–140; horizontal line inside the box represents the median.

The distribution of *S.* *cerevisiae’s* relative abundance (Figure 2b) revealed a bimodal distribution in ASD and TD children. We defined 22% as a cutoff value for the relative abundance of *S.* *cerevisiae* based on abundance pattern and a bimodal distribution. Interestingly, as seen in Figure 2a, children with ASD exhibited a diminished abundance of *S.* *cerevisiae* (<22%) and those children had significantly worse ASD symptoms per the Autism Treatment Evaluation Checklist (ATEC) (*p* = 0.028) scores in comparison to those with a higher abundance of *S.* *cerevisiae* (>22%) (Figure 2d). The distribution of relative abundance of *Candida* (Figure 3a) and of *C.* *albicans* (Figure 3b) also showed a slightly bimodal distribution in ASD children. Based on the C. *albicans* data, we defined a cutoff of 80% relative abundance due to a bimodal distribution from ~ 80% relative abundance (Figure 3a,b). The subset of ASD children with a high relative abundance of *Candida* or *C.* *albicans* (>80%) had significantly higher ATEC scores (worse autism symptoms) than their counterparts (*p* = 0.034 *Candida*; *p* = 0.007 albicans) (Figure 3c,d). It is important to note that higher *Candida* abundance with a high ATEC score was not reported before. Using the same criteria of 80% for *Candida*, ATEC subscores were significantly higher (*p* < 0.05) for sociability (ATEC2), and cognition (ATEC3) (Table 1). Similarly, for *C.* *albicans* (same 80% cutoff), ATEC subscores were significantly higher for sociability (ATEC2), cognition (ATEC3), and additionally for behavior subscores (ATEC4) in ASD children (Table 1). Furthermore, to quantify the total amount of fungi, we performed qPCR targeting the fungal 18S rRNA gene. There was no significant difference in total fungal abundance between TD and ASD (Figure S5).

**Table 1.** Differences in ATEC sub scores based on *Candida* and *C. albicans* abundance.

Category	ATEC for < 80% *Candida*	ATEC for > 80% *Candida*	*p*-values
80% relative abundance threshold for *Candida*			
Speech (ATEC1)	9.72 ± 7.67	9.43 ± 7.16	0.977
Sociability (ATEC2)	14.25 ± 6.60	24.43 ± 5.99	**0.0006**
Cognition (ATEC3)	12.97 ± 6.61	21.57 ± 6.82	**0.007**
Behavior (ATEC4)	25.56 ± 11.98	35.14 ± 14.43	0.08
Total ATEC	62.84 ± 24.78	90.57 ± 25.25	**0.023**
Category	ATEC for < 80% *C.* albicans	ATEC for > 80% *C.* *albicans*	*p*-values
80% relative abundance threshold for *C. albicans*			
Speech (ATEC1)	9.67 ± 7.56	9.67 ± 7.81	0.94
Sociability (ATEC2)	14.30 ± 6.51	25.83 ± 5.15	**0.0001**
Cognition (ATEC3)	13.12 ± 6.57	22.17 ± 7.27	**0.008**
Behavior (ATEC4)	25.12 ± 12.06	39.17 ± 10.68	**0.0102**
Total ATEC	62.55 ± 24.45	96.83 ± 20.87	**0.0037**

The results are presented as mean ± standard deviation. *p* -values were calculated using Mann – Whitney U test. ATEC, Autism Treatment Evaluation Checklist. *p* -values <0.05 are considered statistically significant.

Since we observed a bimodal distribution of fungi in ASD children, with an 80% cutoff for *C.* *albicans* and 22% for *S.* *cerevisiae*, we wanted to confirm whether this distribution was an artifact of the fungi abundance data or if ASD children of this cohort in general are likely to have low *S.* *cerevisiae* and high *Candida*. To investigate this hypothesis, we conducted categorical analyses using the chi-square test and performed a subset analysis using Shannon entropy. The chi-square test showed that children with ASD were significantly more likely to have low *S.* *cerevisiae* (*p* = 0.023), and possibly more likely to have elevated *Candida* (*p* = 0.09) but no significant difference in elevated *C.* *albicans* (*p* = 0.156) compared to TD (Supplementary file S2). To investigate this further, we also performed a subset-specific analysis using absolute sequencing abundance data with 100,000 random permutations. For these three fungi, the null distribution exhibited a bimodal pattern, which is indicative of intrasample heterogeneity. In total, 6 ASD values were identified as belonging to the outlier cohort for *Candida*, and this outlier cohort was deemed to be statistically significant (p-value = 0.00020) (Figure S6). We also observed similar trends for *S.* *cerevisiae* (p-value = 0.00005), and *C.* *albicans* (p-value = 0.00022) (Figure S6). Due to the bimodal distribution of *C.* *albicans* in ASD children, data were in two extreme points, and correlation was not performed with GI and ATEC (Figure S7-S8). However, *S.* *cerevisiae* in ASD children did not exhibit a significant correlation with GI and ATEC scores (Figure S7-S8). See Supplementary file S1 for more information.

## Discussion

The gastrointestinal (GI) tract harbors microbes that are crucial in maintaining host health.^47^ However, mycobiota, which refers to the fungal component of the microbiota, remains under-explored,^47,48^ especially in the context of neurological disorders like ASD, where gut bacteria have been studied widely.^15^ To fill the gaps for mycobiota in ASD, in this pilot study, we sought to elucidate the composition of fungi in children with ASD and analyze the interrelationship between the mycobiota, GI, and ASD symptoms.

Our taxonomic analysis identified a significantly lower abundance of *S. cerevisiae* in children with ASD compared to TD children (Figure 2a). *S*. *cerevisiae* is present in humans, and is considered to be from diet sources, but a genetically closely related strain *S. cerevisiae var. boulardii*, acts as a probiotic, conferring protection against antibiotics or *C. difficile* induced diarrhea and ameliorating conditions such as gastroenteritis,^49–51^ and ulcerative colitis.^52^ Kobliner *et al*. reported that a single case treatment with *S. cerevisiae var. boulardii* to a child with ASD who also had gastrointestinal dysfunction, obsessive-compulsive disorder (OCD) and self-injurious behavior (SIB), and *S. boulardii* treatment successfully reduced OCD and SIB symptoms.^53^ Conversely, Zou *et al*. reported that *S. cerevisiae* relative abundance was significantly higher in ASD compared to TD children, in contrast to the results in this study,^32^ suggesting *S. cerevisiae* abundance in ASD could be strain or cohort-specific.

Interestingly, we observed children with ASD with < 22% of the abundance of *S. cerevisiae* had significantly worse autism symptoms (higher ATEC scores) (Figure 2d) compared to children with ASD with > 22% of the abundance of *S. cerevisiae*, which suggests that *S. cerevisiae* could have some protective function and be beneficial to reduce some autism symptoms.^53^ Whereas *S. cerevisiae* was the dominant fungi in TD children, *Candida* and *C. albicans* were dominant in the gastrointestinal flora of ASD children (Figure 1b, S4). Nonetheless, no statistically significant difference was observed between TD and ASD children for *Candida* (Table S2) and *C. albicans* (Figure 2c) using univariate analysis. However, the chi-square test showed a trend that children with ASD were likely to have higher (*p* = 0.09) *Candida* than TD. *Candida* (yeast) is an opportunistic pathogen and commonly found in the human gut. However, an increased abundance of fungi (primarily *C. albicans*) has been reported in children with ASD.^4, 30–34^ Looking closely at the data, we observed a bimodal distribution of *Candida* and *C. albicans* abundance within ASD children. TD had only 5.2% of *Candida* and *C. albicans* (2 out of 38 TD) but 17.5% of ASD children (7 out of 40 ASD) with an elevated abundance of *Candida* (>80%) and 15% of ASD children (6 out of 40 ASD) with a higher abundance of *C. albicans* (>80%) had significantly higher ATEC scores (including sociability, cognition, and behavior) (Figure 3c,d, Table 1), which suggests that overgrowth of *Candida* and *C. albicans* and lower abundance of *S. cerevisiae* could be linked to worse autism symptoms (Figure 2A,D). Most of the previous studies have not investigated the possible relationship of fungal species to ASD symptoms, except for Alookaran *et al*.^33^ which did not find a relationship, perhaps because it was under-powered.^4,30–34^ While the relative abundance of *Candida* and *C. albicans* did not exhibit a significant difference between the ASD and TD cohorts, a subset analysis using Shannon entropy of ASD samples for *Candida*, *C. albicans* and *S. cerevisiae* exhibited a unique subpopulation (Figure S6), with a significant difference. ^32^Strati *et al*.^34^ found that the relative abundance of *Candida* was more than double in ASD compared to TD children, although a bimodal distribution was not described in that cohort. Previous studies *via* semi-quantitative yeast culture of fecal microbiota also showed that ASD children exhibited higher *Candida* culture positives than TD children.^4,31^ This trend has also been reported for conditions like irritable bowel syndrome (IBS)^54^ and Crohn’s Disease.^55^

Furthermore, one study^4^ found that the ASD group with positive yeast cultures had a 48% lower level of secretory Immunoglobulin A (sIgA) compared to the ASD group with negative yeast cultures, *p* = 0.09 (not statistically significant), which is the primary antibody against bacteria and fungi in the mucous membranes.^42,43^ Even though these findings were not statistically significant, they suggest the depletion of sIgA may be due to the production of IgA-specific proteases secreted by *C. albicans*, which degrade sIgA study.^4,56–58^ Interestingly, for cellular growth, *Candida* or *C. albicans* catabolizes amino acids and increases the production of ammonia,^59^ which interacts with gut propionic acid, and leads to the conversion of beta-alanine.^60^ Due to structural similarities to gamma-aminobutyric acid (GABA, C_4_H_9_NO_2_), beta-alanine (C_3_H_9_NO_2_) crosses the blood-brain barrier, acting as a partial GABA antagonist. This partial blockade of GABA receptor sites stimulates increased production of GABA, an inhibitory neurotransmitter, in the brain in pursuit of achieving homeostatic balance. Consequently, the overproduction of GABA may play a partial role in explaining the manifestation of autistic behaviors.^61–63^ *Candida* also produces mycotoxins, such as alcohols, aldehydes, 2-phenylethanol, tartaric acid, isoamyl alcohol, and tryptophol,^36,37,39,41^ which are known to affect cognitive function adversely.^40^ It is important to note that in some studies, ASD children with a higher abundance of *Candida* suffer from GI issues.^31^ In this study, we observed that GI issues were significantly higher in ASD children than in TD (Figure 1a), and significantly moderate-positively correlated with ATEC scores (Figure S1). However, a correlation was not performed due to the extreme data points of *C. albicans* with GI symptoms (Figure S8B), and the 6-GSI index was not associated with fungal abundance. This suggests that elevated levels of fungi or their metabolites may affect primarily neurological symptoms but not GI symptoms. It has been reported that children with ASD often use more oral antibiotics in infancy,^10,28^ which may foster the proliferation of *Candida*. Intriguingly, anti-fungal interventions have been identified as having therapeutic potential in ASD treatment.^41,44,60,62^ Altogether, the above findings suggest that a subset of children with ASD have a very high abundance of *Candida* (primarily *C*. *albicans)* which could be linked to worse ASD symptoms.^60,62^

*S. cerevisiae* and *Candida* were the most dominant fungi in this cohort, but it is important to note that it is common to observe the fungi’s relative abundance data with dominance of 1–2 taxa.^32,34^ Along with *C. albicans* and *S. cerevisiae*, we also observed *Penicillium*, *Candida sake*, *Cladosporium*, *Parastagonospora nodorum*, and *Cyberlindnera jadinii* as some of the most abundant mycobiota in this cohort (Figure 1b); however, none of these mycobiota showed a significant difference between ASD *vs* TD children (Table S2). *Penicillium* (TD = 3.75E–02 ± 1.68E–01 vs. ASD = 1.79E–03 ± 4.13E–03, *p* = 0.48, Table S2) is one of the common gut mycobiota in healthy humans and associated with a vegetarian diet^64^ and animal-based diet.^65^ *Candida sake* (TD = 1.88E–02 ± 9.08E–02 vs. ASD = 2.40E–02 ± 1.52E–01, *p* = 0.29, Table S2) has been reported in healthy individual’s feces.^66,67^ *Cladosporium* (TD = 5.05E–03 ± 1.61E–02 vs. ASD = 3.59E–02 ± 1.59E–01, *p* = 0.34, Table S2) have been found in the healthy individual’s gut^67,68^ as well as in adult patients with mild cognitive impairment (MCI) compared with controls.^69^ *Parastagonospora nodorum* (TD = 4.72E–05 ± 1.41E–04 vs. ASD = 3.93E–02 ± 1.72E–01, *p* = 0.25, Table S2) is a plant pathogen,^70^ not been explored well in the human gut, and one possible reason of its presence in feces could be that this fungus might have passed through food to these individuals. *Cyberlindnera jadinii* (TD = 8.39E–03 ± 4.50E–02 vs. ASD = 6.71E–02 ± 2.22E–01, *p* = 0.71, Table S2) have also been found in adult patients with MCI compared with controls.^69^

On the other hand, ASD has also been found to be linked with sex-specific bias^3^ favoring males over females with a 4:1 ratio^71^ and it has been hypothesized that males could be more susceptible to mycotoxins and environmental changes than females.^3^ However, in this study, we did not have enough data and sample size on gender ratio to evaluate gender bias with respect to fungal abundance.

A limitation of this study is the small sample size to distinguish the fungi community between ASD and TD cohorts, as well as their interrelation with autism and GI symptoms. This study is based on fungal ITS gene amplicon sequencing, but shotgun metagenomics could shed light on understanding the functional dynamics of the fungal microbiome in ASD children. In summary, fungal ITS gene amplicon sequencing revealed that S. *cerevisiae* was significantly lower in ASD vs. TD controls. Autism severity (ATEC scores) was significantly higher in the small subset of ASD children with higher *Candida* (7 out of 40 ASD; 17.5%), and *C. albicans* abundance (6 out of 40 ASD; 12.2%). However, GI symptoms did not correlate with an abundance of *S. cerevisiae* or *C. albicans*, suggesting that *Candida* or *C*. *albicans* may affect neurological/ASD symptoms but not GI symptoms. Similarly, Alookaran *et al.*, also did not observe a correlation between GI symptoms and fungal microbiota in ASD children. This lack of GI symptoms correlation, suggests that because of the lack of obvious symptoms fungi infections can remain in the gut without noticeable GI symptoms and thus be untreated for a long time.^33^

We hypothesize that *Candida* may exacerbate neurological symptoms in ASD children and early detection of fungi infection/dysbiosis, along with treatment using antifungals targeting *Candida* and *C. albicans*, may help reduce autism severity or at least some core symptoms. We also recommend an intervention study with antifungals and possibly implementing an anti-yeast diet to determine possible safety and efficacy. Future research with a larger cohort, a longitudinal approach with an intervention, targeted qPCR for *Candida* and *C. albicans*, and shotgun metagenomic analyses, is warranted to understand the functional role of fungi in ASD children.

## Methods

### Enrollment of study participants

A total of 78 participants, encompassing 38 TD children and 40 children with ASD, were enrolled in this study. The age range of the subjects was 3–17 years, with similar ages for both groups, and with both groups being primarily male (Table S1). A critical inclusion criterion was that the subjects did not use any type of antibiotic or antifungal medications for a minimum duration of one month before fecal sample collection. GI symptoms were assessed utilizing a modified 6-item version^4^ of the original Gastrointestinal Severity Index (GSI) questionnaire with 6 items of GI severity index.^72^ As in previous publications,^4,10^ high-GI symptoms are defined as a 6-GSI score above 3.^4^ Autism symptoms were assessed with the Autism Treatment Evaluation Checklist (ATEC) to corroborate the diagnosis of ASD. We also evaluated nutritional supplements and special diets, such as gluten-free/casein-free (GF/CF) diet, probiotics use, seafood consumption, and nutrient supplements (e.g., vitamins/calcium).^18^ For more information, see the supplementary file S1-S2.

### Ethical and institutional review board information

The study was conducted according to the guidelines of the Declaration of Helsinki and approved by the Institutional Review Board at Arizona State University, AZ, USA (ASU IRB Protocol #: 1206007979, 1004005109). The study was advertised in Arizona, USA. Interested candidates were mailed a consent form (with a preliminary questionnaire and GI severity index scoring) and general participation questions. Upon receiving a signed informed consent form and completed questionnaires from parents/guardians, sample collection kits were mailed to the participants.

### Sample collection and fecal DNA extraction

Parents/guardians collected fecal samples from their children and immediately froze them. Subsequently, these samples were shipped to Arizona State University with dry ice. Upon receipt at the laboratory, samples were stored at −80°C until DNA extraction. Total genomic DNA was isolated using a PowerSoil DNA extraction kit (Mobio Carlsbad, CA) with a default protocol from the manufacturer.

### Fungal ITS gene amplicon sequencing

The extracted fecal DNA was processed for fungal sequencing using the fungal internal transcribed spacer (ITS) Illumina Amplicon Protocol obtained from the Earth Microbiome Project (http://www.earthmicrobiome.org/protocols-and-standards/its/). The library was constructed using the ITS1f-ITS2 primer set (Forward primer 5’-CTTGGTCATTTAGAGGAAGTAA-3’ and Reverse primer 5’-GCTGCGTTCTTCATCGATGC-3’). Amplicons were subsequently pooled and subjected to purification using the MoBio UltraClean PCR Clean-Up Kit. Finally, an aliquot from the pooled sample was sequenced using an Illumina MiSeq sequencer with single-end forward reads of ~ 250bp. Sequencing was performed at the Genomics core in the Biodesign Institute of Arizona State University, AZ, USA.

### Sequencing data analyses

Raw sequencing reads were processed using QIIME2 (v2022.02).^45^ Pre-processing involved removing the PCR primers, DNA barcodes, and the filtration of sequence reads for low-quality nucleotides. Reads were filtered based on Phred score > 25 and trimmed at 210bp. Denoising was performed using DADA2^73^ to remove duplicate reads and chimeric sequences. Amplicon sequence variants (ASV) were assigned using the UNITE database (v8, 10.5.2021)^46^ The sequences were clustered at a 99% similarity against the UNITE database. Rarefaction was performed at 1,329 reads depth to consider all samples for further downstream analysis. Downstream analyses and visualization of relative abundance and diversity indices were performed using the Dokdo API (https://github.com/sbslee/dokdo, v1.14.0) in Python with Qiime2.

### Subset analysis using Shannon entropy of *Candida, C.* *albicans* and *S.* *cerevisiae*

Shannon entropy is defined as a measure of the uncertainty in a random variable or a set of data, quantifying the level of surprise or unpredictability in its outcomes.^74^ This metric serves as a useful tool for evaluating the diversity or uniformity inherent in a sample dataset. One prominent application of Shannon entropy is subpopulation detection, as this technique can be used to identify sample subsets exhibiting unique behaviors.^75^ Lower entropy values within these subsets often indicate the presence of potential subpopulations characterized by distinct or heightened homogeneity. Finally, the significance of entropic differences can be quantified by comparing the actual entropy values examined for a subgroup against that of comparably sized permutations.

*Shannon entropy, as it is commonly formulated in the context of information theory:*

*H(X) = −∑iP(X = xi) ⋅ log2(P(X = xi))*

*H(X) represents the Shannon entropy of the random variable X.*

*xi represents each possible outcome of the random variable.*

Subset analysis was conducted on fungi taxa exhibiting bimodal distributions. ASD and TD samples exceeding 2 standard deviations from the median value were assigned to be included in a clinical condition-specific outlier subset. The Shannon entropy of this subset was subsequently determined. A null distribution of Shannon entropy values was established by 100,000 random permutations, and subsequently, p-values were calculated (see supplementary file S1 for more details).

### Quantitative PCR (qPCR) for the total fungal abundance

To quantify the total fungal DNA in fecal DNA, a TaqMan qPCR assay was performed using FungiQuant universal primers FungiQuant-F: 5′-GGRAAACTCACCAGGTCCAG-3′ and FungiQuant-R: 5′-GSWCTATCCCCAKCACGA3′, FungiQuant-Prb(6FAM) 5′-TGGTGCATGGCCGTT-3′ (MGBNFQ)^76^ and QuantStudio3 instrument (Applied Biosystems, CA, USA). We used the following qPCR conditions: 10 min at 95°C for Taq activation, 15 s at 95°C for denaturation, and 1 min at 65°C for annealing and extension x 50 cycles.^76^ All samples and controls (positive and negative) were run in triplicates, and *C. albicans* (ATCC 5314) genomic DNA (0.37 pg/μL) was used as a standard control to generate calibration curves. Total fungal DNA was reported in gene copy number/gm wet feces. Due to constraints in DNA availability, qPCR was conducted on a subset of the cohort, comprising 28 TD and 20 ASD children.

### Statistical analysis and plots

To investigate and compare the differences in fungal composition (as determined by both sequencing and qPCR), and symptoms between TD and ASD groups, a nonparametric statistical test, the Mann-Whitney U test, was performed, assuming the data were non-normally distributed due to a small sample size. To avoid false positives for taxonomic data, the false discovery rate (FDR) was determined for each significant taxa (*p* < 0.05) using a leave-one-out approach, and *p* < 0.05 was considered statistically significant.^12,77,78^ Additionally, Pearson and Spearman correlation tests were performed to analyze correlations between taxa and symptoms, and unadjusted *p*-values <0.05 were considered statistically significant. The chi-square test was performed for categorical analysis between ASD and TD groups for *Candida*, *C. albicans* and *S*. *cerevisiae*. For the box plot of *C. albicans* and *S. cerevisiae*, a pseudo-count (0.0001) was incorporated for each sample due to zeros (Figure 2a,c), and subsequently, data was transformed into a log10 scale. All statistical analyses were performed in R (v0.4.0), and figures were generated through R using *ggplot2* (v3.4.2), *ggpubr* (v0.6.0), and OriginPro (v2023b). Inkscape (v1.1) was used to create or edit the figures.

## List of abbreviations

ASD: Autism spectrum disorders
ASU: Arizona State University
ATEC: Autism Treatment Effectiveness Checklist
DNA: Deoxyribonucleic acid
CDC: Centers for Disease Control and Prevention
GI: Gastrointestinal
GSI: Gastrointestinal Severity Index
ITS: Internal transcribed spacer
sIgA: secretory Immunoglobulin A
PCR: Polymerase Chain Reaction
QC: Quality Control
qPCR: quantitative Polymerase Chain Reaction
Qiime: Quantitative Insights Into Microbial Ecology
RNA: Ribonucleic acid
TD: Typically-Developing

## Supplementary Material

Supplemental Material

Supplemental Material

## Data Availability

The data presented in this study are openly available in the NCBI SRA repository under BioProject ID PRJNA993699 and can be accessed at https://www.ncbi.nlm.nih.gov/bioproject/993699.
