# Supplementary material for: Bimodal distribution of intestinal Candida in children with autism and its potential link with worse ASD symptoms
Source: Gut Microbes Rep. 2024 Jun 27;1(1):2358324. doi: 10.1080/29933935.2024.2358324 (PMC12940154; doi:10.1080/29933935.2024.2358324)
Supplement: Supplemental Material [file KGMR_A_2358324_SM9578.docx]

**Bimodal distribution of intestinal *Candida* in children with autism and its potential link with worse ASD symptoms**

Khemlal Nirmalkar^1*^, Jigar Patel^1^, Dae-Wook Kang^1#^, Andrew Bellinghiere^1^, Devin A. Bowes^1^, Fatir Qureshi^2^, James B. Adams^1,3^, Rosa Krajmalnik-Brown^1,4*^

^1^Biodesign Center for Health Through Microbiomes, Arizona State University, Tempe, AZ, USA

^2^Whitehead Institute for Biomedical Research, Cambridge, MA, 02142, USA

^3^School for Engineering of Matter, Transport, and Energy, Arizona State University, Tempe, AZ 85287, USA

^4^School of Sustainable Engineering and the Built Environment, Arizona State University, Tempe, AZ 85281, USA

^#^Current address: Department of Civil and Environmental Engineering, University of Toledo, Toledo, OH 43606, USA

^*^Correspondence: Rosa Krajmalnik-Brown [Dr.Rosy@asu.edu](mailto:Dr.Rosy@asu.edu) and Khemlal Nirmalkar [khem@asu.edu](mailto:khem@asu.edu)

**Supplementary Materials S1**

**Methods**

Participant Recruitment and Demographic Features: A total of seventy-eight (78) participants were enrolled in the study, comprising 38 typically developing (TD) and 40 children with autism spectrum disorders (ASD). The inclusion criteria necessitated participants to be within the age bracket of 2.5 to 17 years, and devoid of exposure to any class of antibiotic or antifungal pharmaceutical agents within the preceding 30 days. TDs who were consanguineous first-degree relatives of individuals diagnosed with ASD were precluded from participation. The Autism Treatment Evaluation Checklist (ATEC) was employed and encompasses four dimensions: 1) verbal communication/language, 2) social interaction aptitude, 3) sensory processing/cognitive acuity, and 4) health and physical comportment. The ATEC composite score is calculated by aggregating the scores across these four domains. Elevated ATEC scores are indicative of the heightened severity of ASD.

Additionally, gastrointestinal symptoms were evaluated employing an adapted version of the Gastro-Intestinal Severity Index (GSI) questionnaire. ^1,2^ Of the GSI dimensions, six symptom categories were incorporated: constipation, diarrhea, stool consistency, stool odor, flatulence, and abdominal discomfort. A trichotomous rating scale was utilized for each symptom category, and an aggregate score was computed, termed the 6-GI Severity Index (6-GSI). Excluded dimensions encompassed ‘unaccounted daytime irritability’, ‘nocturnal arousal’, and ‘abdominal sensitivity’.

**Subset analysis**

A null distribution of Shannon entropy values was established by randomly permuting the same sample size as the outlier cohort from the entire distribution of values. Entropy was computed for each randomly selected subset, thus creating a null distribution. In total, 100,000 permutations were performed. Using the relationship between the values present in the null distribution relative to that of the outliers, the statistical significance of the subset differing from the complete population from which they were sampled could be assessed. A p-value was calculated based on this proportion of values in the null distribution that were as extreme or more than that of the outlier cohort. A small p-value less than 0.001 indicated that the observed difference in Shannon entropy was statistically significant, suggesting a meaningful difference between the identified outlier cohort and the general sample population.

**Steps/algorithm for the permutation test**

*1. Compute the observed values of the test statistic*

*t_obs_ = T(X1,…,Xm,Y1,…,Y_n._*

*2. Randomly permute the data. Computer the statistic again using the permutated data.*

*3. Repeat the previous step B times and let T1,…,Tb denote the resulting values.*

*4. The approximate p-value is*

$$\frac{1}{B} \sum_{j=1}^{B} I(T_{jn}>t_{obs} f)$$

**Results:**

### Correlation between ASD Symptoms with C. albicans, S. cerevisiae

### Due to the bimodal distribution of C. albicans, data were in two extreme points, making a correlation with ATEC scores (Fig. S7A) and with S. cerevisiae (Fig. S7C), with GI symptoms (Fig. S8B) in ASD children not statistically appropriate; therefore, a linear regression was not performed for these data sets. Comparing S. cerevisiae with ATEC scores (Fig. S7B), and GI symptoms (Fig. S8A) for ASD children and S. cerevisiae with GI symptoms in TD’s (Fig. S8C) did not demonstrate a significant correlation. For TD children, data points for GI symptoms with C. albicans (Fig. S8D) and between S. cerevisiae and C. albicans (Fig. S8E) were also in two extreme points, and correlation analysis was not performed.

**Tables:**

| **Table S1.** Characteristics of study participants | | |
| --- | --- | --- |
| **Category** | **TD** | **ASD** |
| Total Subjects (n) | 38 | 40 |
| Male/Female | 29 / 9 | 37 / 3 |
| Age (years) | 8.24 ± 3.78 | 8.65 ± 3.93 |
| ATEC | N/A | 67.82 ± 26.79 |
| PDD-BI | N/A | -55.13 ± 60.24 |
| 6-GSI | 1.11 ± 1.69 | 4.7 ± 2.61 |
| Data are shown with mean ± standard deviation. TD - Typically Developing, ASD - Autism Spectrum Disorder, ATEC - Autism Treatment Evaluation Checklist, PDD-BI - Pervasive Developmental Disorder Behavior Inventory, GSI - Gastrointestinal Severity Index, N/A - Not Applicable. | | |

| **Table S2: Univariate comparison of fungal taxa (most abundant) between TD and ASD children.** | | | |
| --- | --- | --- | --- |
| **Name** | **TD** | **ASD** | **p-values** |
| Total *Candida* | 8.77E-02 ± 2.22E-01 | 2.13E-01 ± 3.83E-01 | 0.83 |
| Non-*Candida*_Total_fungi | 9.12E-01 ± 2.22E-01 | 7.87E-01 ± 3.83E-01 | 0.83 |
| Non-*C.albicans*_Total_fungi | 9.36E-01 ± 2.09E-01 | 8.37E-01 ± 3.59E-01 | 0.67 |
| Non-*C.albicans*_Total_*Candida* | 2.32E-02 ± 9.28E-02 | 5.04E-02 ± 1.84E-01 | 0.64 |
| *s_Saccharomyces_cerevisiae* | 5.19E-01 ± 4.19E-01 | 2.91E-01 ± 3.69E-01 | **0.011** |
| *s_Candida_albicans* | 6.45E-02 ± 2.09E-01 | 1.63E-01 ± 3.59E-01 | 0.67 |
| *g_Penicillium* | 3.75E-02 ± 1.68E-01 | 1.79E-03 ± 4.13E-03 | 0.48 |
| *s_Candida_sake* | 1.88E-02 ± 9.08E-02 | 2.40E-02 ± 1.52E-01 | 0.29 |
| *g_Cladosporium* | 5.05E-03 ± 1.61E-02 | 3.59E-02 ± 1.59E-01 | 0.34 |
| *s_Parastagonospora_nodorum* | 4.72E-05 ± 1.41E-04 | 3.93E-02 ± 1.72E-01 | 0.25 |
| *s_Cyberlindnera_jadinii* | 8.39E-03 ± 4.50E-02 | 6.71E-02 ± 2.22E-01 | 0.71 |
| *s_Penicillium_carneum* | 1.21E-02 ± 7.08E-02 | 1.87E-02 ± 1.18E-01 | 0.60 |
| *s_Candida_tropicalis* | 4.37E-03 ± 2.28E-02 | 2.64E-02 ± 1.09E-01 | 0.51 |
| *s_Aspergillus_heterocaryoticus* | 1.56E-02 ± 5.19E-02 | 1.62E-02 ± 9.78E-02 | 0.052 |
| *s_Issatchenkia_orientalis* | 2.44E-02 ± 1.04E-01 | 3.58E-03 ± 2.04E-02 | 0.40 |
| *g_Wallemia* | 2.25E-02 ± 1.37E-01 | 6.94E-03 ± 3.93E-02 | 0.74 |
| *s_Dekkera_bruxellensis* | 1.70E-03 ± 4.18E-03 | 2.82E-02 ± 1.49E-01 | 0.77 |
| *o_Saccharomycetales* | 2.17E-02 ± 8.84E-02 | 9.00E-03 ± 4.92E-02 | 0.64 |
| *f_Sclerotiniaceae* | 1.09E-02 ± 6.16E-02 | 3.66E-04 ± 2.16E-03 | 0.66 |
| *s_Alternaria_angustiovoidea* | 2.29E-02 ± 1.33E-01 | 1.60E-02 ± 5.05E-02 | 0.26 |
| *s_Candida_vartiovaarae* | 0.00E+00 ± 0.00E+00 | 1.70E-05 ± 1.08E-04 | 0.34 |
| *s_Candida_ethanolica* | 1.66E-05 ± 8.30E-05 | 0.00E+00 ± 0.00E+00 | 0.14 |
| In this table, the Mann-Whitney U test was used to compare fungal taxa between TD and ASD children. Data are shown as mean±standard deviation. p<0.05 was considered statistically significant. TD - typically developing, ASD - autism spectrum disorder, s - species, g - genera, o - order, f -family. | | | |


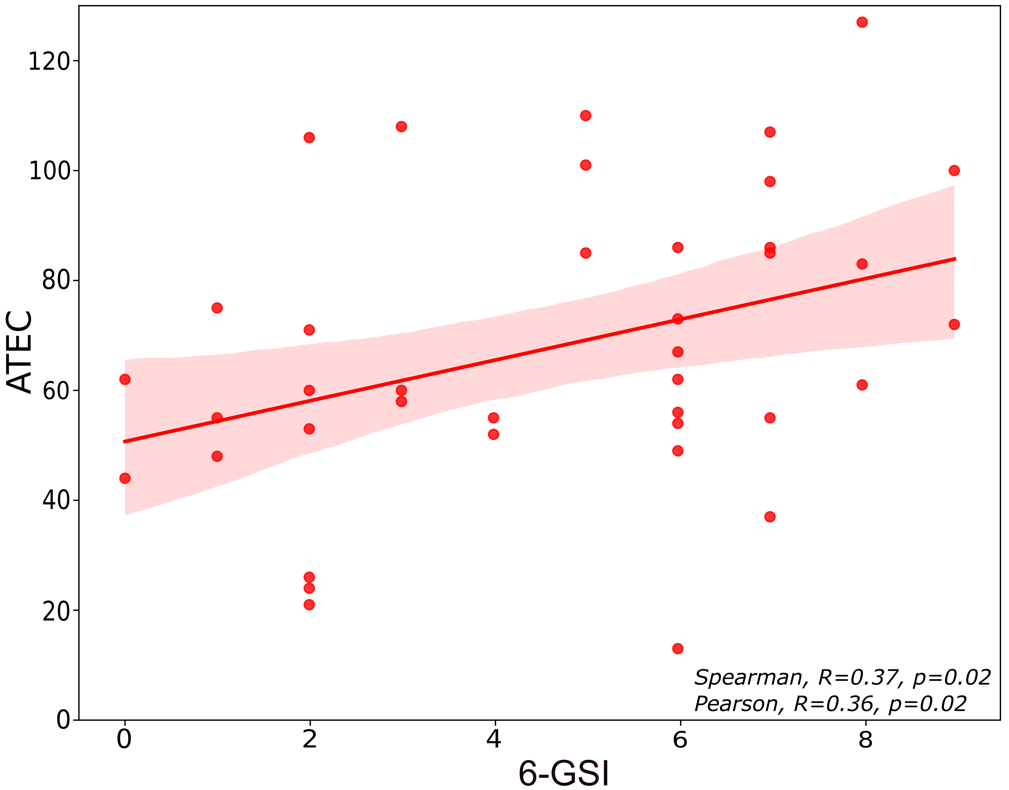


Figure S1: Correlation analyses between ATEC scores and 6-GSI scores in children with ASD. ATEC - Autism Treatment Evaluation Checklist, 6-GSI - 6-Gastrointestinal Severity Index. *p*-values < 0.05 are considered statistically significant.


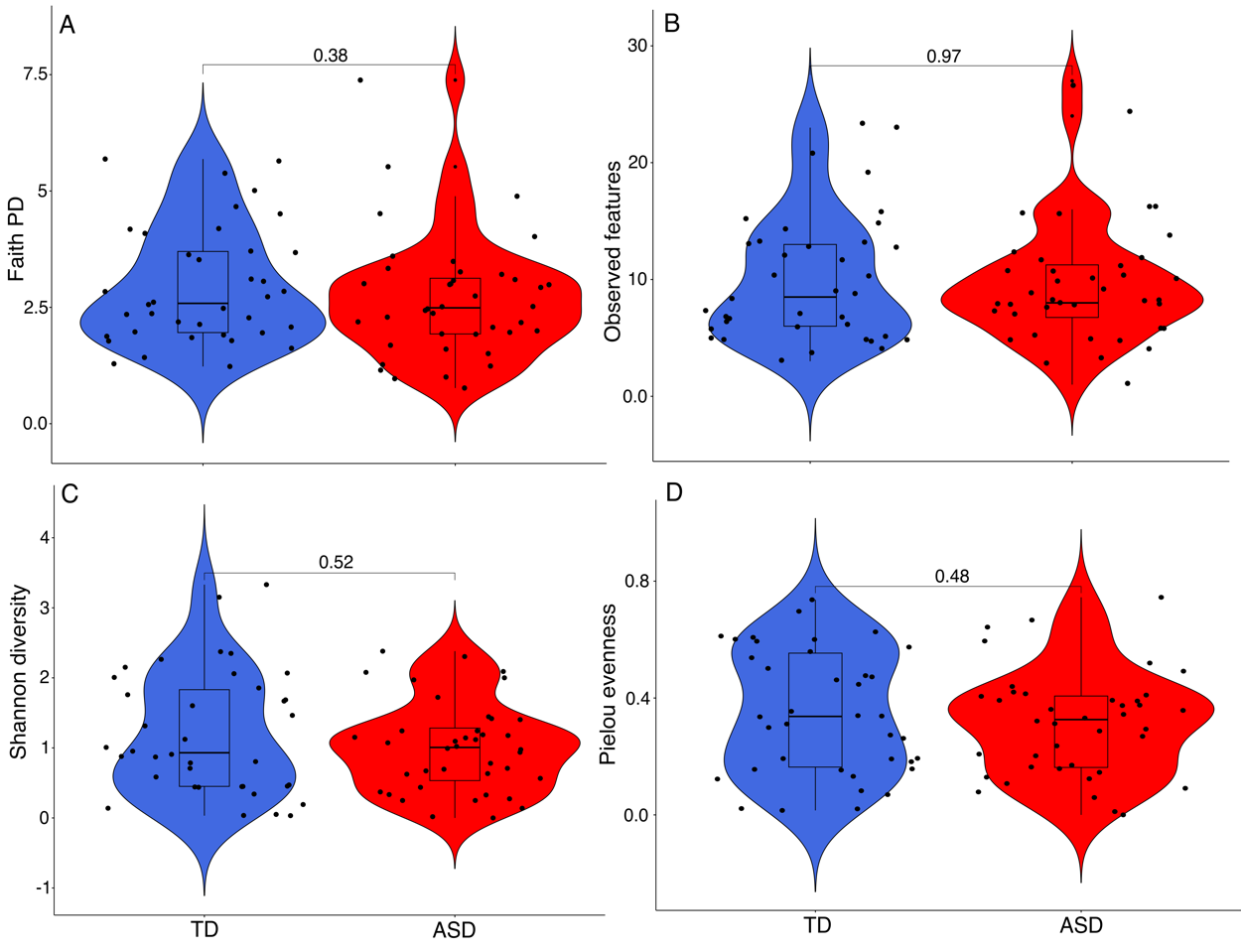


Figure S2: Alpha‐diversity indices Faith PD (A), observed features (B), Shannon diversity (C) and Pielou Evenness (D) comparisons between TD and ASD children. Blue color represents TD and red color for ASD participants. *p* values < 0.05 are considered statistically significant. ASD: autism spectrum disorder; TD: typically developing. The horizontal line inside the box represents the median.


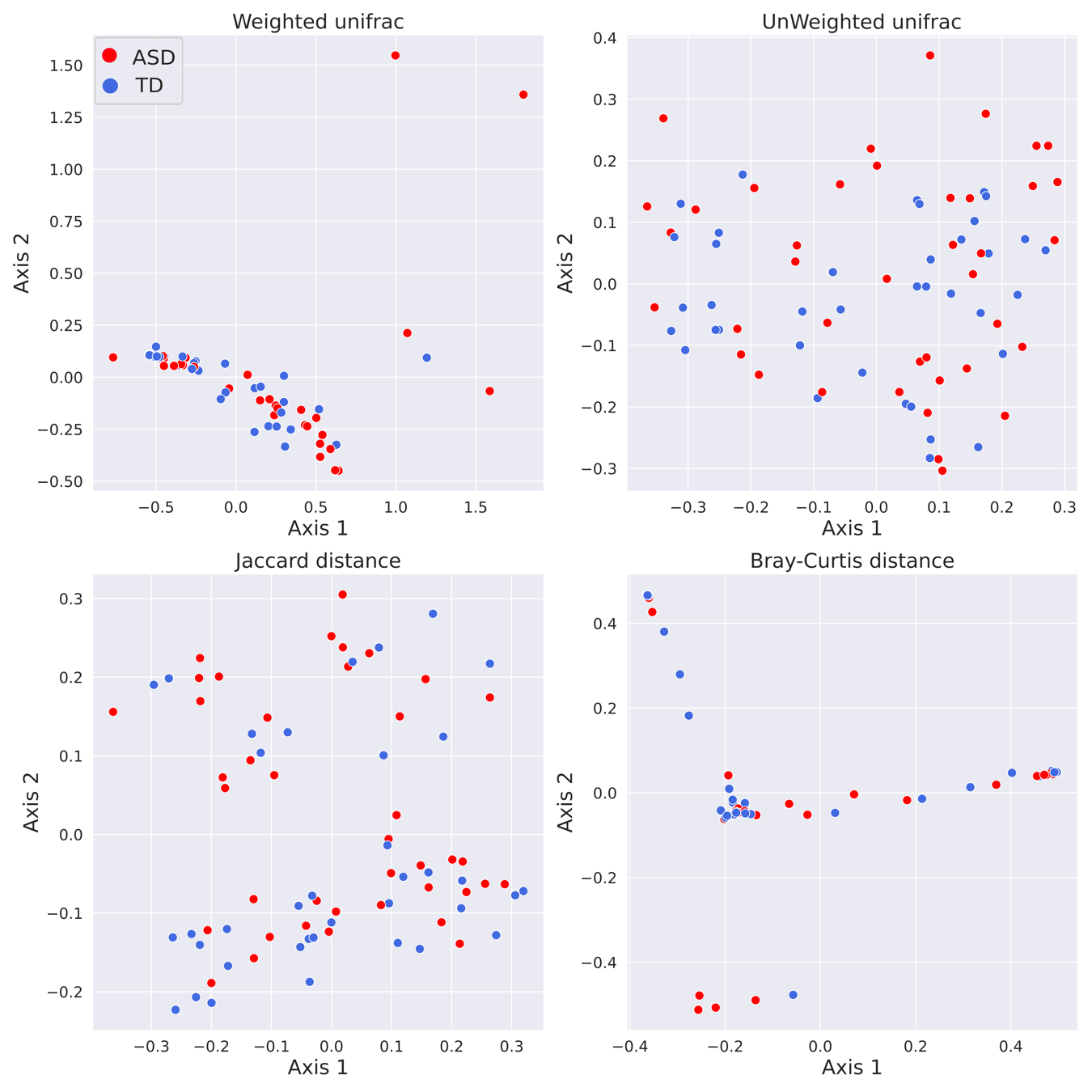


Figure S3: 2D beta‐diversity indices Weighted Unifrac (A), Unweighted Unifrac (B), Jaccard distance (C) and Bray-Curtis distance (D) comparisons between TD and ASD children. Blue color represents TD and red color for ASD participants. ASD: autism spectrum disorder; TD: typically developing.


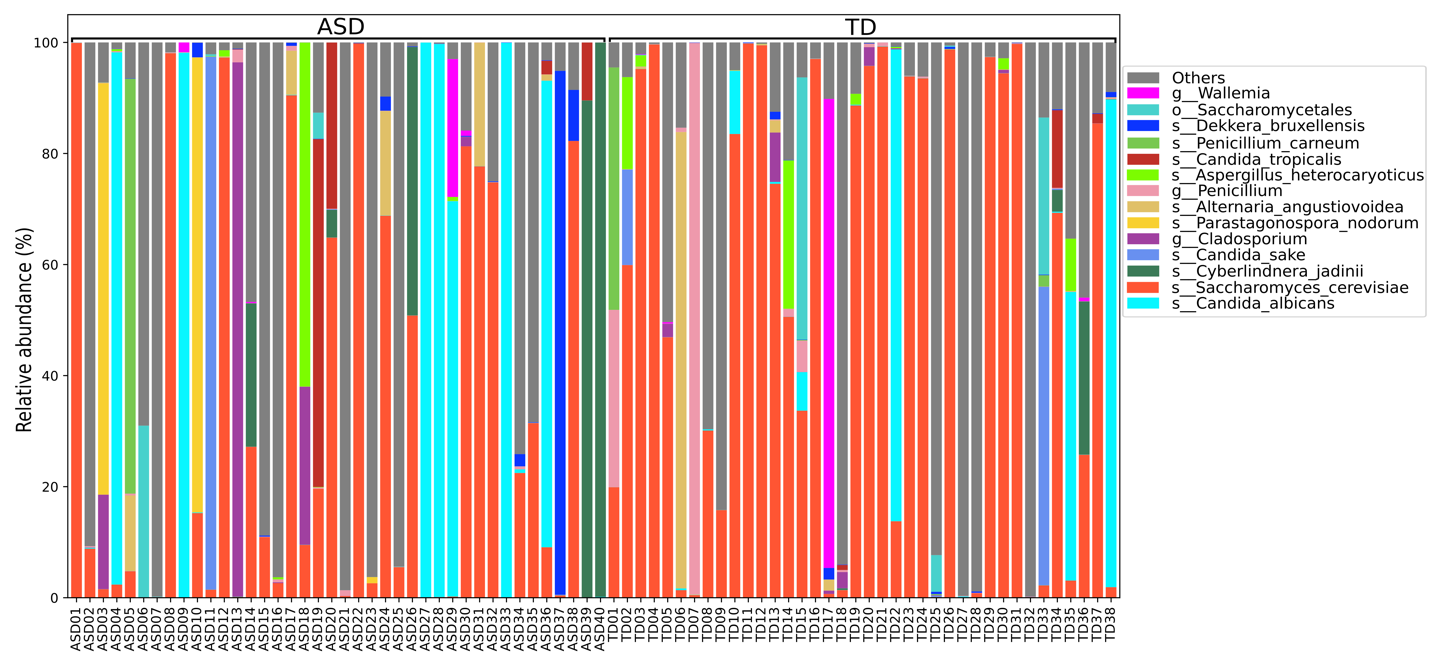


Figure S4: Average relative abundance (%) of Top 15 fungal taxa in TD and children with ASD. TD- typically developing; ASD- autism spectrum disorder. Average relative abundance of fungi in each participant, refer to Figure 1B. o-order; f-family; g-genera; s-species.


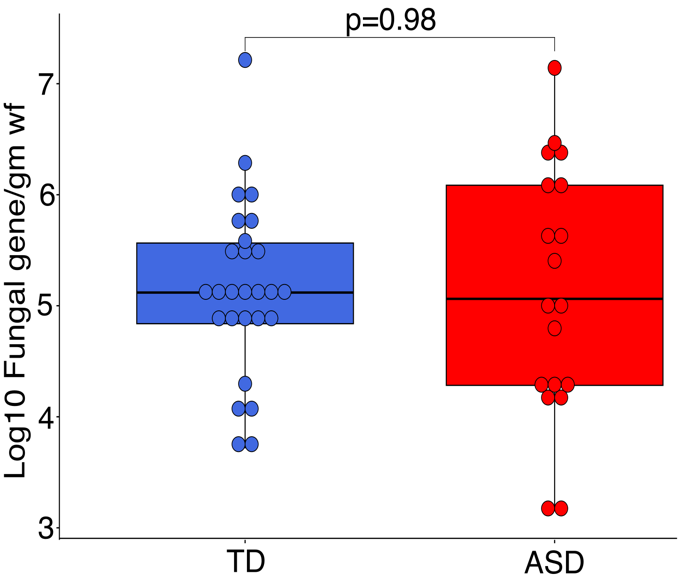


Figure S5: Quantitative measurement (qPCR) of total fungal 18S rRNA gene (log10) copy/gm wet feces in TD and ASD children. Blue color represents TD, and red color for ASD participants. p-values < 0.05 are considered statistically significant. ASD: autism spectrum disorder; TD: typically developing; wf: wet feces. The horizontal line inside the box represents the median.


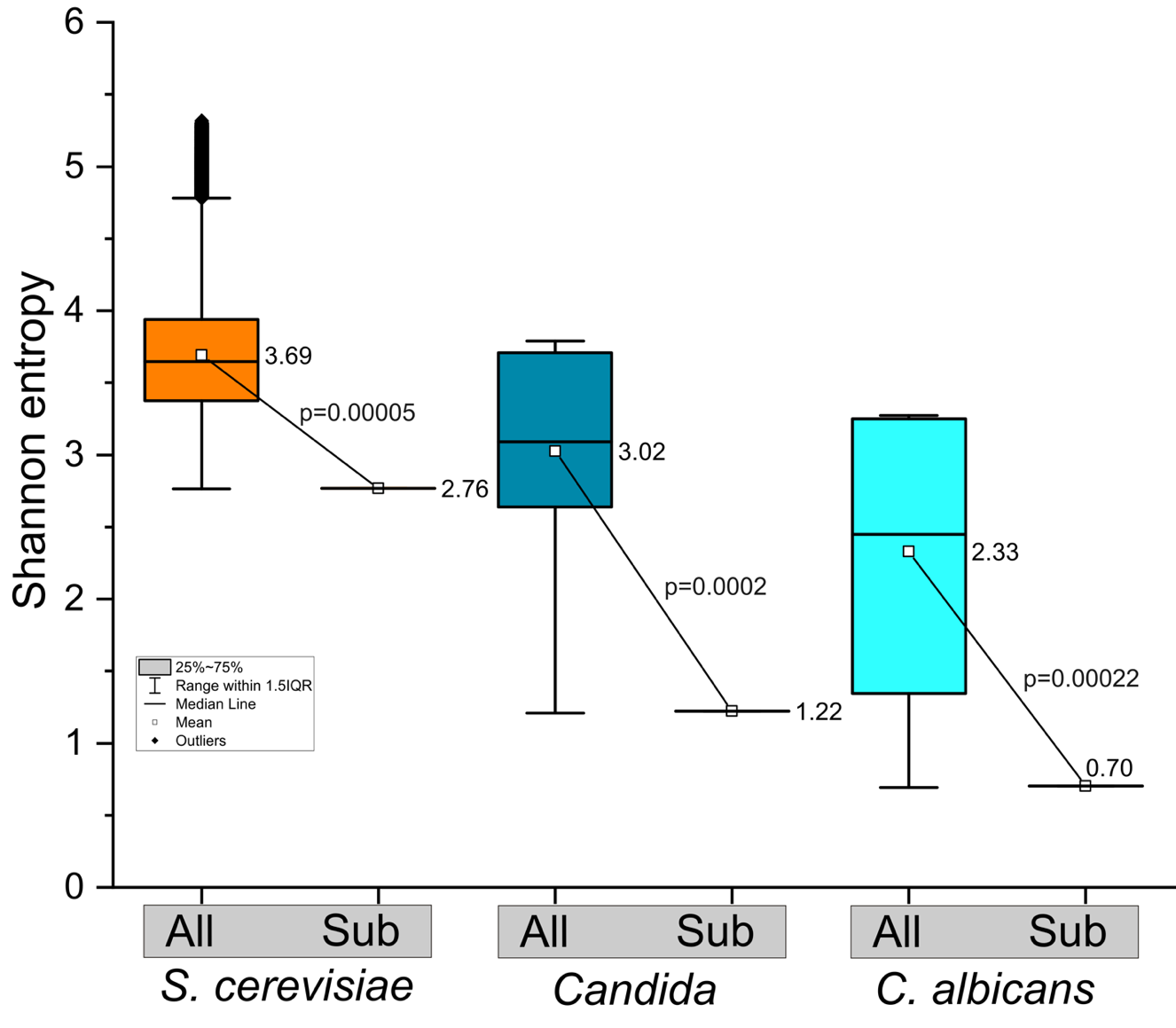


Figure S6: Difference in Shannon entropy between 100,000 random permutations of outlier-sized sample sets collected from *S. cerevisiae, Candida* and *C. albicans* against complete sample distribution. All = complete set of samples (ASD+TD); Sub = 6-subset of ASD samples. p-values < 0.05 are considered statistically significant. ASD: autism spectrum disorder; TD: typically developing.


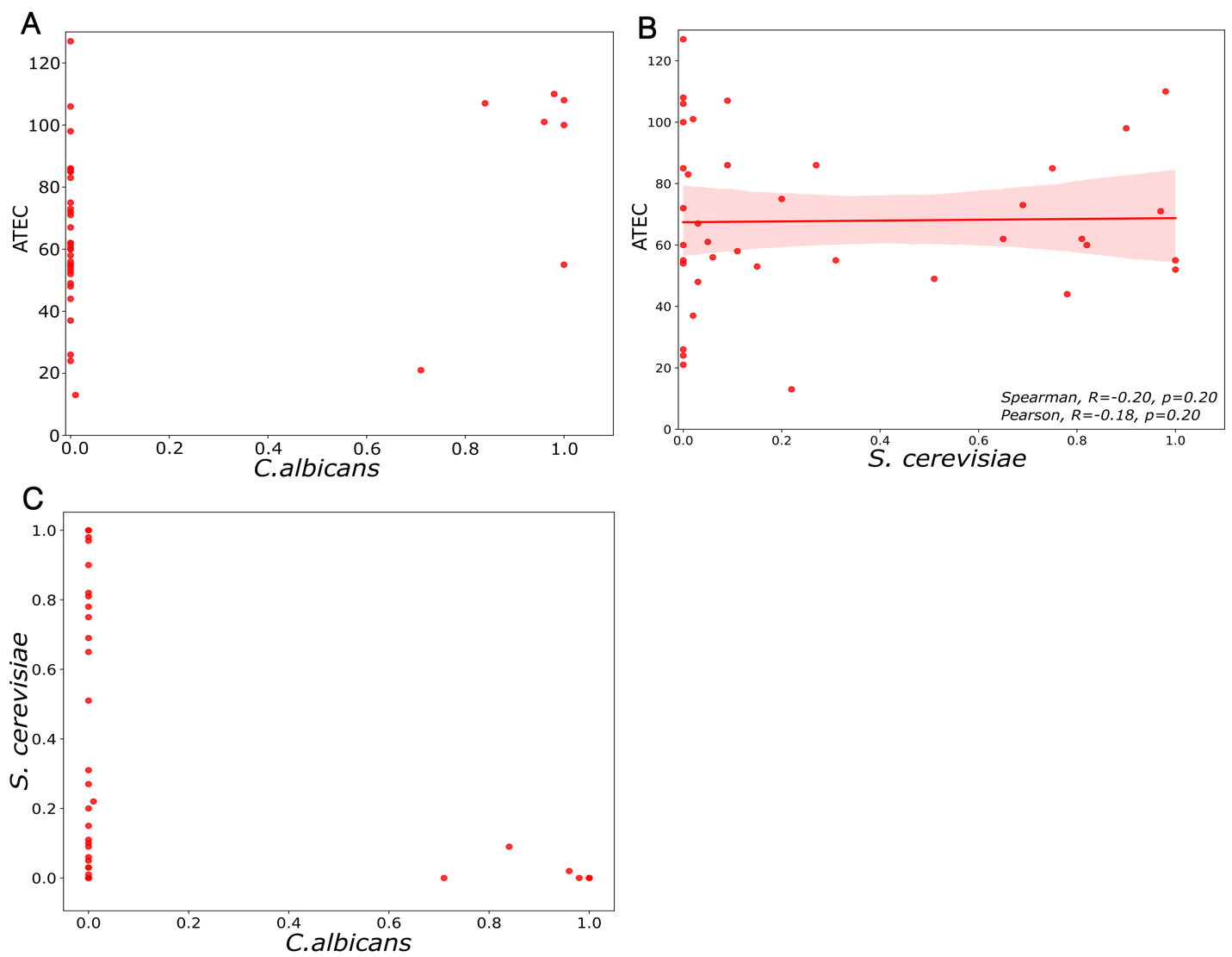


Figure S7: Data distribution between fungal taxa and symptoms in children with ASD. Between the relative abundance of *C. albicans* and ATEC (A), *S. cerevisiae* and ATEC (B), between the relative abundance of *C. albicans* and *S. cerevisiae* (C). Due to extreme data points and bimodal distribution, linear regression was not performed for (A) and (C). p values < 0.05 are considered statistically significant.


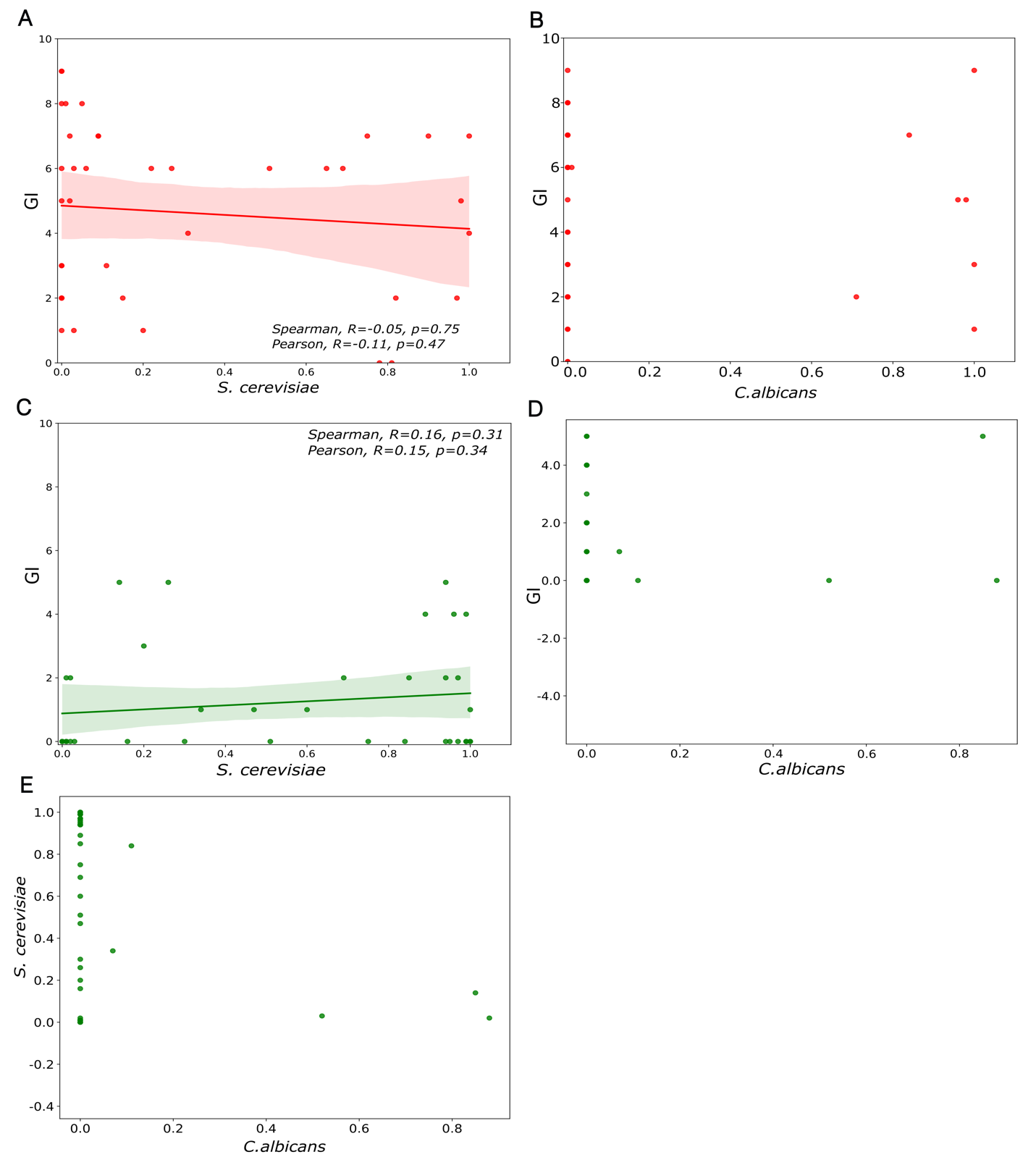


Figure S8: Data distribution between fungal taxa and GI symptoms in TD and ASD children. Correlation between GI scores and *S. cerevisiae* (A), and between GI scores and *C. albicans* in ASD children (B), between GI scores and *S. cerevisiae* (C), between GI scores and *C. albicans* (D), and between *C. albicans* and *S. cerevisiae* in TD children (E). Due to extreme data points and bimodal distribution, linear regression was not performed for (B), (D) and (E). GI - 6-GSI - 6-Gastrointestinal Severity Index (GSI), *p* values < 0.05 are considered statistically significant.

References

1. Adams JB, Johansen LJ, Powell LD, Quig D, Rubin RA. Gastrointestinal flora and gastrointestinal status in children with autism - comparisons to typical children and correlation with autism severity. BMC Gastroenterol 2011; 11.

2. Schneider CK, Melmed RD, Barstow LE, Enriquez FJ, Ranger-Moore J, Ostrem JA. Oral human immunoglobulin for children with autism and gastrointestinal dysfunction: A prospective, open-label study. J Autism Dev Disord 2006; 36:1053–64.
